# Diverse functions of the inward-rectifying potassium channel Kir5.1 and its relationship with human diseases

**DOI:** 10.3389/fphys.2023.1127893

**Published:** 2023-02-27

**Authors:** Chaojie Zhang, Jia Guo

**Affiliations:** ^1^ Nephrology Research Center, The First Affiliated Hospital of Zhengzhou University, Zhengzhou, China; ^2^ Institute of Nephrology, Zhengzhou University, Zhengzhou, China; ^3^ Henan Province Research Center for Kidney Disease, Zhengzhou, China; ^4^ Key Laboratory of Precision Diagnosis and Treatment for Chronic Kidney Disease in Henan Province, Zhengzhou, China

**Keywords:** cancer, deafness, Kir5.1, potassium channel, respiration

## Abstract

The inward-rectifying potassium channel subunit Kir5.1, encoded by *Kcnj16*, can form functional heteromeric channels (Kir4.1/5.1 and Kir4.2/5.1) with Kir4.1 (encoded by *Kcnj10*) or Kir4.2 (encoded by *Kcnj15*). It is expressed in the kidneys, pancreas, thyroid, brain, and other organs. Although Kir5.1 cannot form functional homomeric channels in most cases, an increasing number of studies in recent years have found that the functions of this subunit should not be underestimated. Kir5.1 can confer intracellular pH sensitivity to Kir4.1/5.1 channels, which can act as extracellular potassium sensors in the renal distal convoluted tubule segment. This segment plays an important role in maintaining potassium and acid-base balances. This review summarizes the various pathophysiological processes involved in Kir5.1 and the expression changes of Kir5.1 as a differentially expressed gene in various cancers, as well as describing several other disease phenotypes caused by Kir5.1 dysfunction.

## 1 Introduction

**FIGURE 1:**
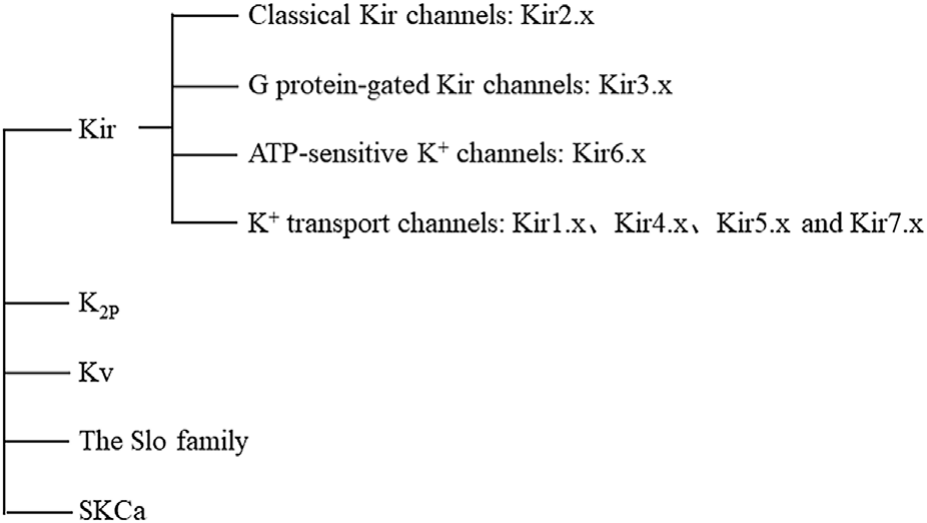
Classification of potassium channels. Abbreviations: Kir, inward rectifiers; K_2P_, four transmembrane segments with two pores; Kv, voltage-gated; Slo, *slowpoke*; SKCa, the Ca^2+^ -activated SK family (small conductance calcium activated potassium channels); ATP, adenosine-triphosphate.

Ion channels, a type of transmembrane protein, are key pathways for ions to enter and exit cells. Diseases caused by the dysfunction of ion channels in cell and organelle membranes are called to channelopathies (Kim, 2014), which have been found underlie many human diseases, such as epilepsy, arrhythmia, asthma, and diabetes (Kass, 2005; Ashcroft, 2006; Valverde et al., 2011). Among different kinds of ion channels, potassium channels play a decisive role in various physiological processes such as maintenance of the cell resting membrane potential and regulation of hormone secretion and blood pressure. According to their different structures and functions, potassium ion channels can be mainly divided into five categories: 1) inward rectifiers, Kir; 2) four transmembrane segments-2 pores, K_2P_; 3) voltage-gated, Kv; 4) the Slo (*slowpoke*) family; and 5) the Ca^2+^-activated SK family (small conductance calcium activated potassium channels), SKCa (Figure 1) (Gonzalez et al., 2012).

Kir channels are indispensable in maintaining cell resting membrane potential, regulating cell excitability, and regulating systemic electrolyte homeostasis. Inward-rectifying Kir channels exhibit the ability to allow potassium ions to pass through them that increases with hyperpolarization and decreases with depolarization. This characteristic is not an inherent characteristic of channel proteins, but requires the participation of intracellular substances, such as Mg^2+^ and polyamines (Hibino et al., 2010).17 Kir subunits encoded by *KCNJ* genes have been identified, and functional Kir channels consist of four subunits in a homologous or heterologous manner (Manis et al., 2021). There are seven Kir channel subfamilies (Kir1.x-Kir7.x) that can be divided into four groups according to their function: 1) classical Kir channels (activated by phosphatidylinositol 4,5-bisphosphate, PtdIns(4,5)P2): Kir2.x; 2) G protein-gated Kir channels (regulated by G protein-coupled receptors): Kir3.x; 3) ATP-sensitive K^+^ channels (regulated by intracellular nucleotides and by various pharmacological agents): Kir6.x; and 4) K^+^ transport channels (affected by intracellular pH): Kir1.x, Kir4.x, Kir5.x, and Kir7.x (Hibino et al., 2010).

**FIGURE 2:**
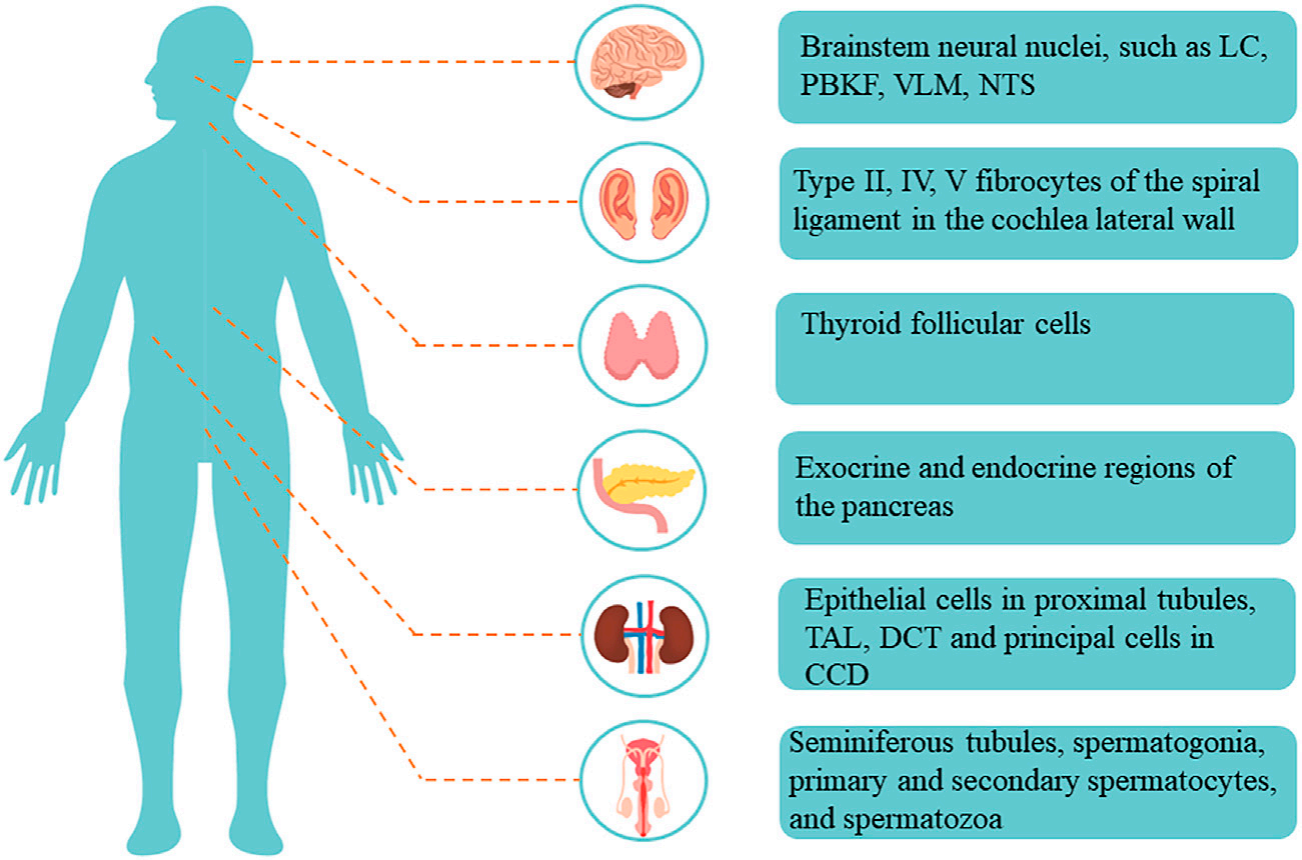
Distribution and localization of Kir5.1 in the human body. Abbreviations: LC, locus coeruleus; PBKF, parabrachial, and Kolliker-Fuse; VLM, ventrolateral medullary; NTS, nucleus of solitary tract; TAL, thick ascending limb; DCT, distal convoluted tubule; CCD, cortical collecting duct.

Among them, Kir5.1, encoded by *KCNJ16*, is expressed in many organs and tissues, such as the brain, kidney, pancreas, thyroid, and testis (Figure 2) (Liu et al., 2000; Hibino et al., 2004a; Hibino et al., 2004b; Wu et al., 2004). Depending on its location, it performs different functions. Recent studies have demonstrated the important role of Kir5.1 channels in different human organs and diseases; for example, *KCNJ16* biallelic mutations can lead to salt wasting, disturbed acid-base homeostasis, and sensorineural deafness, (Welling, 2016; Manis et al., 2020; Schlingmann et al., 2021). With the development of researches on Kir5.1, it has been found that Kir5.1 is closely related to the phenotypes of various diseases, such as participating in the formation of salt-sensitive hypertension, affecting respiratory compensatory ability and reproductive ability (Palygin et al., 2017a; Puissant et al., 2019; Zhao et al., 2021). In addition, *KCNJ16* has not only been found to be a differentially expressed gene in a variety of cancers, but also *KCNJ16* gene mutation is associated with sudden infant death syndrome, Brugada syndrome, lymphoma and other diseases (Juang et al., 2014; Daino et al., 2019). Even more impressive, Kir5.1 was previously thought to be unable to form functional potassium channels alone. However, studies have found that SQM, the carboxy-terminal amino acid sequence of Kir5.1, can bind to the PZD3-SH3 domain of postsynaptic density protein 95 (PSD-95) to form a functional Kir5.1 channel in brain neurons. As a potential target of the protein kinase A (PKA)-mediated signaling pathway, Kir5.1/PSD-95 channels may play an important functional role in synaptic transmission (Tanemoto et al., 2002).

In view of the new findings regarding the role of Kir5.1 in human diseases, we believe that it still has many important functions worth further investigation. This review introduces the inward-rectifying potassium channel Kir5.1 from three aspects: the main physiological characteristics, the pathological processes involved and the connection with various human diseases. Overall, this review broadly summarizes the functions of Kir5.1 reported in existing studies, and provides new ideas for future research exploring the diverse biological functions of Kir5.1 and its possible significance in human diseases (Zhang et al., 2021).

## 2 Main physiological characteristics of Kir5.1

### 2.1 Intracellular pH sensitivity of Kir5.1

In 2000, Liu et al. found the new inward-rectifying potassium channel subunit Kir5.1 (encoded by *KCNJ16*) by searching the Genbank database. It was found that human *KCNJ16*, localized on chromosome 17q25, and mouse *Kcnj16*, localized on the distal region of chromosome 11, were highly homologous. Further tissue distribution studies showed that human *KCNJ16* was significantly expressed in human renal tubular epithelial cells, pancreatic acinar cells, ductal cells, and the thyroid gland (Liu et al., 2000). Previous studies suggested that Kir5.1 could not form functional homomeric channels, but could form functional heteromeric channels (Kir4.1/5.1 and Kir4.2/5.1) with Kir4.1, encoded by *Kcnj10*, or Kir4.2, encoded by *Kcnj15* (Pessia et al., 2001). By transfecting Kir4.1 alone and co-transfecting Kir4.1 and Kir5.1 in HEK293T cells, researchers found that the involvement of the Kir5.1 subunit conferred intracellular pH (pHi) sensitivity to Kir4.1/5.1 channels. Kir4.1 channel activity remained stable between pHi value was 6–8. When the pHi value was 7, Kir4.1/5.1 channel activity decreased to 20% of that when the pHi value was 7.3, and when the pHi value was 6–6.5, channel activity almost disappeared. In addition, Kir4.1/5.1 could also be activated by intracellular alkalinization. Changing the extracellular pH in this range had no effect on either Kir channel (Tanemoto et al., 2000). However, compared with Kir4.2 channels, the participation of Kir5.1 did not significantly increase the sensitivity of Kir4.2/5.1 to pHi, because of the pH sensing mechanism at the C-terminal of the Kir4.2 subunit making it significantly more sensitive to pHi than the Kir4.1 channel (Pessia et al., 2001). The pHi sensitivity of the Kir4.1/5.1 and Kir4.2/5.1 channels and their localization in renal tubular epithelial cells suggest that they may play an important role in regulating acid-base and electrolyte balances in the kidney.

### 2.2 Potassium sensor function of Kir5.1

Kir4.1/5.1 is not only the main potassium channel in the basolateral membrane of mouse kidney distal convoluted tubule (DCT) epithelial cells, but is also the extracellular potassium sensor, which determines the potassium conductance of the basolateral membrane, forms the transmembrane potential of tubular epithelial cells, senses the change of extracellular K^+^ concentration, and changes the membrane potential and intracellular Cl^−^ concentration of tubular epithelial cells (Zhang et al., 2014; Palmer, 2015; Su et al., 2019; Wu et al., 2019). In the renal DCT, NaCl cotransporter (NCC) is mainly responsible for regulating Na^+^ and Cl^−^ reabsorption and K^+^ secretion in distal tubules. Kir4.1/5.1 regulates NCC activity through the with-no-lysine kinase (WNK)-STE20/SPS-1-related proline-alanine-rich protein kinase (SPAK) pathway. Finally, it regulates salt reabsorption and K^+^ secretion in distal tubules and collecting ducts (Terker et al., 2015; Hadchouel et al., 2016; Welling, 2016). For example, in hypokalemia, increased Kir4.1/5.1 activity hyperpolarizes cell membranes, drives intracellular Cl^−^ efflux, causes low Cl^−^ levels in epithelial cells, further activates WNK, activates NCC through the phosphorylation of SPAK, increases sodium reabsorption in the DCT, reduces K^+^ excretion in distal tubules and collecting ducts, and maintains blood potassium levels. The opposite is true for hyperkalemia (Terker et al., 2015; Welling, 2016). Previous studies have shown that knockout of Kir4.1 can eliminate the effect of dietary potassium on NCC activity (Cuevas et al., 2017; Wang et al., 2018). In addition, Wu et al. found that in wild-type mice a high-potassium diet could inhibit the expression of NCC, whereas a low-potassium diet could promote the expression of NCC. This phenomenon was eliminated in Kir5.1 knockout mice, which had a decreased ability to expel K^+^ on a high-potassium diet and to retain K^+^ on a low-potassium diet (Wu et al., 2019). Therefore, knockout of Kir5.1 also abolished the effect of dietary potassium on NCC activity. This suggests that Kir5.1, like Kir4.1, is an indispensable part of the Kir4.1/5.1 channel for its function as a potassium sensor in the DCT. In conclusion, Kir4.1/5.1 regulates NCC activity by sensing changes in extracellular K^+^ concentration and altering basolateral membrane conductance to ensure that urinary potassium excretion matches dietary potassium intake.

## 3 Pathophysiological processes related to Kir5.1

### 3.1 Kir5.1 dysfunction can cause metabolic acidosis and hypokalemia

In the kidney of rats and mice, Kir5.1 is mainly expressed in the basolateral membrane of epithelial cells in the thick ascending limb (TAL), the DCT, and principal cells in the cortical collecting duct (CCD) (Tucker et al., 2000; Lourdel et al., 2002; Lachheb et al., 2008). Kir5.1 is also expressed in proximal tubule (Tucker et al., 2000; Schlingmann et al., 2021). Although there is no research shown that Kir5.1 is localized in the basolateral membrane of the proximal tubule epithelial cells, the expression pattern of Kir5.1 should be consistent throughout the renal tubular segment. Besides, both Kir4.2 and Kir4.1, which bind to Kir5.1 to form functional potassium channels, are localized in the basolateral membrane, and if Kir5.1 is expressed in the apical membrane of the proximal tubule, it is likely to be non-functional (Lourdel et al., 2002; Bignon et al., 2020). Therefore, we suggest that Kir5.1 is mainly expressed in the basolateral membrane of the proximal tubule epithelial cells. In addition, in immunoperoxidase staining of the rat kidney, Kir5.1 expression is also observed in endothelial cells around glomerular capillaries (Tucker et al., 2000).

Previous studies have demonstrated that Kir4.1/5.1 is the main potassium channel in the basolateral membrane of DCT epithelial cells, contributes to K^+^ circulation with Na^+^-K^+^-ATPase, and participates in regulating systemic electrolyte balance and maintaining acid-base homeostasis (Palygin et al., 2017b; Palygin et al., 2018; Lin et al., 2022; Wang and Lin, 2022). Among them, mutations in the *KCNJ10* gene encoding the Kir4.1 subunit can cause EAST/SeSAME syndrome (epilepsy, ataxia, sensorineural deafness, tubulopathy/seizures, sensorineural deafness, ataxia, intellectual disability, and electrolyte imbalance), which manifests as epilepsy, ataxia, sensorineural deafness, hypokalemia, and metabolic alkalosis (Bockenhauer et al., 2009; Reichold et al., 2010). In contrast to metabolic alkalosis caused by *KCNJ10* gene mutation, mice with a targeted disruption of *Kcnj16* and Dahl salt-sensitive (Dahl SS) rats with *Kcnj16* gene mutations showed distinct hyperchloremic acidosis. Moreover, urinary ammonium excretion was reduced in mice with targeted disruption of *Kcnj16* (Paulais et al., 2011; Puissant et al., 2019). A similar phenomenon has been found in human studies: in 2021, Schlingmann et al. found that eight patients with *KCNJ16* biallelic variants at different locations had common clinical features, including salt wasting, acid-base homeostasis disorder, sensorineural deafness, and decreased urinary ammonium excretion, and all but one presented with metabolic acidosis (Schlingmann et al., 2021; Webb et al., 2021). The symptoms of Kir5.1 dysfunction are different from those of Kir4.1 dysfunction, suggesting that Kir5.1 in the kidney is not limited to forming Kir4.1/5.1 channels with Kir4.1 in distal tubules, but also plays other important potential roles in which Kir4.1 is not involved.

In addition, Kir5.1 also plays an essential role in the proximal tubule. The proximal tubule is not only the main reabsorption site of Na^+^, K^+^, Cl^−^, and HCO_3_^−^, but is also the main secretion site of H^+^ and NH_4_^+^. To explore the function of Kir5.1 in proximal tubules, Schlingmann et al. further analyzed the expression of *Kcnj16*, *Kcnj10*, and *Kcnj15* mRNA in the mouse kidney. The results showed that *Kcnj16* was widely expressed in renal tubules, whereas *Kcnj10* was mostly distributed in the distal tubule, and *Kcnj15* was mainly distributed in the proximal tubule (Schlingmann et al., 2021). Interestingly, *Kcnj15*-deleted mice exhibits hyperchloremic acidosis, decreased bicarbonate reabsorption threshold, and urinary NH_4_^+^ excretion dysfunction (Bignon et al., 2020). Moreover, previous studies have shown that Kir4.2 expression is significantly reduced in Kir5.1 knockout mice (Wu et al., 2019). Therefore, *Kcnj16* gene mutations may cause the depolarization of proximal tubule epithelial cells through the dysfunction of Kir4.2/5.1 channels and decreased Kir4.2 expression, leading to reduced Na^+^-HCO_3_^−^ cotransporter NBCe1 (encoded by *SLC4A4*) transport in the basolateral membrane. The accumulation of HCO_3_^−^ in epithelial cells leads to intracellular alkalinization, which can cause abnormal expression levels of glutamine transporters in the basolateral membrane and intracellular ammoniagenic enzymes, and eventually lead to decreased ammonia production (Lee et al., 2018). In Kir5.1 dysfunctional individuals, these mechanisms lead to a phenomenon similar to that observed in *Kcnj15*-deleted mice, that is, the decreased ability of proximal tubules to reabsorb bicarbonate and secrete NH_4_^+^, resulting in metabolic acidosis and decreased urinary ammonium excretion levels, which patients with *KCNJ10* mutations do not have (Paulais et al., 2011; Schlingmann et al., 2021). These findings reveal a non-redundant role of Kir5.1 in the proximal tubule. Notably, there is no aminoaciduria or glycosuria in either the *Kcnj15* or *Kcnj16* gene variants, suggesting that Kir4.2 or Kir5.1 dysfunction caused a phenomenon similar to the permanent forms of familial isolated proximal renal tubular acidosis. Bicarbonate reabsorption disorder and low NH_4_^+^ secretion occurred in the absence of extensive proximal tubule reabsorption disorder, suggesting that potassium channels other than Kir4.2 or Kir4.2/5.1 may exist in the proximal tubule to maintain basolateral membrane K^+^ circulation, thus maintaining normal material reabsorption in the apical membrane of tubular epithelial cells (Bignon et al., 2020; Schlingmann et al., 2021). Interestingly, one patient in this cohort presented with metabolic alkalosis, and further studies revealed that the *KCNJ16* mutant in this patient had a strong inhibition of Kir4.1 and milder effect on the Kir4.2/5.1. Therefore, we speculate that whether the *KCNJ16* mutant shows metabolic acidosis or metabolic alkalosis depends on whether the effect of the mutant on Kir4.2/5.1 and Kir4.2 in the proximal tubule is dominant or the effect on Kir4.1/5.1 and Kir4.1 in the distal tubule is dominant (Schlingmann et al., 2021).

Another significant manifestation caused by Kir5.1 dysfunction in the kidney is hypokalemia (Paulais et al., 2011; Palygin et al., 2017a; Puissant et al., 2019; Schlingmann et al., 2021). In the body, potassium balance not only requires Na^+^-K^+^ pumping and Na^+^-H^+^ exchange to change the distribution of intracellular and extracellular fluid, but also requires regulation of potassium excretion by changing the transmembrane potential of renal tubular epithelial cells and the flow rate of distal tubular fluid. Although Kir4.1 and Kir5.1 are both essential for the function of the Kir4.1/5.1 potassium sensor and both cause hypokalemia after knockout, they have different mechanisms (Reichold et al., 2010). *KCNJ10* gene mutation can reduce the potassium conductance of the basolateral membrane of DCT epithelial cells, inactivate the WNK–SPAK–NCC pathway, reduce NCC reabsorption, increase the flow rate of distal tubule fluid, quickly remove K^+^ secreted into the tubule fluid from distal tubules and collecting ducts, and greatly reduce the concentration of K^+^ in the tubule. The driving force for the secretion of K^+^ into tubule fluid in tubular epithelial cells increases, leading to a decrease in blood K^+^ (Zhang et al., 2014). However, in mice with targeted disruption of *Kcnj16*, owing to the presence of Kir4.1 channels in the basolateral membrane of DCT cells, the activity of NCC after Kir5.1 dysfunction does not decrease, but in fact increases, in response to the increase of Kir4.1 channel conductance (Paulais et al., 2011; Wu et al., 2019). Therefore, the occurrence of hypokalemia in mice with Kir5.1 dysfunction may be related to the widespread distribution of Kir5.1 in renal tubules; Kir4.2/5.1 is expressed in the basolateral membrane of proximal tubular epithelial cells, and Kir4.1/5.1 is expressed in the basolateral membrane of TAL and DCT epithelial cells and CCD principal cells. Kir5.1 dysfunction causes a large range of K^+^ reabsorption disorders in the basolateral membrane of tubular epithelial cells, resulting in hypokalemia. These studies suggest that Kir5.1 plays an important role in regulating systemic electrolyte homeostasis in the kidney.

### 3.2 Kir5.1 dysfunction causes loss of salt-sensitive phenotype in Dahl SS rats

Previous studies have found that dietary potassium deficiency can increase blood pressure and salt sensitivity (elevated blood pressure in response to high salt intake) by changing cell membrane voltage, leading to hyperpolarization and stimulating NCC activation (Terker et al., 2015). This is closely related to the potassium sensor function of Kir4.1/5.1. Kir4.1/5.1 can perceive the change of extracellular K^+^ concentration, change cell membrane potential, and regulate NCC activity to coordinate urinary potassium excretion and dietary potassium intake under normal conditions. When dietary potassium deficiency occurs, the concentration of extracellular K^+^ decreases, and Kir4.1/5.1 hyperpolarizes the cell membrane and causes NCC activation through the WNK-SPAK-NCC pathway to limit potassium loss, sometimes even at the cost of increasing blood pressure (Terker et al., 2015; Welling, 2016).

Dahl SS rats are a genetically stable and widely used model of hypertension, and their salt sensitivity is characterized by a significant increase in blood pressure after a high-salt diet (Wadei and Textor, 2012). Palygin et al. found that Kir5.1 expression was significantly increased in SS rat kidneys after a high-salt diet, whereas Dahl SS rats with *Kcnj16* knockout (SS^Kcnj16−/−^) showed hypotension, and their blood pressure did not increase after a high-salt diet. This suggests that the Kir5.1 channel plays a key role in the development of salt-sensitive hypertension (Palygin et al., 2017a). Thus, targeting Kir5.1 may be an effective way to lower blood pressure during the development of salt-sensitive hypertension.

In addition, SS^Kcnj16−/−^ rats also show significant salt consumption, which is not seen in mice with targeted disruption of *Kcnj16* (Paulais et al., 2011; Palygin et al., 2017a; Wu et al., 2019). Renal DCT reabsorption not only requires K^+^ circulation in the basolateral membrane to provide a Na^+^ concentration gradient favorable for reabsorption in the apical membrane of epithelial cells, but also requires normal NCC activity. This difference in salt consumption may be owing to the different localization of Kir4.1 in the renal tubular epithelial cells of the two knockout animals, although Kir4.1 expression is upregulated in both. In SS^Kcnj16−/−^ rats, Kir4.1 is significantly expressed in the cytoplasm, with only a small amount in the basolateral membrane, in contrast to SS rats, where the Kir4.1 channels are located in the basolateral membrane. However, Kir4.1 is significantly upregulated in the basolateral membrane of mice with targeted disruption of *Kcnj16* (Paulais et al., 2011; Palygin et al., 2017a). Kir4.1 in the basolateral membrane can maintain K^+^ circulation and upregulate the expression of NCC in mice with targeted disruption of *Kcnj16*, thus maintaining normal salt reabsorption. However, in SS^Kcnj16−/−^ rats, although the expression of NCC is upregulated, only a small amount of Kir4.1 is present in the basolateral membrane, which is insufficient to maintain normal K^+^ circulation; this disrupts the Na^+^ concentration gradient in the apical membrane of epithelial cells, reduces the reabsorption of the DCT, and thus leads to salt consumption (Palygin et al., 2017a). The different localization of Kir4.1 in the kidneys of the two animals with Kir5.1 dysfunction may be owing to their different gene backgrounds (Wu et al., 2019). Furthermore, knockout of *Kcnj16* markedly alters the regulation and function of the renin-angiotensin-aldosterone system in Dahl SS rats (Manis et al., 2019). Therefore, as an essential component of potassium sensor and K^+^ circulation as well as an important regulator of renin-angiotensin-aldosterone system, Kir5.1 plays a key role in the development of salt-sensitive hypertension.

### 3.3 Kir5.1 dysfunction reduces respiratory compensatory ability against hypercapnia and hypoxia

Arterial CO_2_ controls respiratory activity in mammals by acting on peripheral respiratory chemoreceptors in the carotid body and aortic body, as well as central respiratory chemoreceptors located in the brainstem. Central respiratory chemoreceptors can drive respiratory activity to be closely linked to arterial pH and CO_2_ levels, a process thought to be regulated by some PCO_2_/pH-sensitive channels (Puissant et al., 2019). Studies have found that, in addition to pHi sensitivity, Kir4.1/5.1 channels are also highly sensitive to CO_2_ (Tucker et al., 2000; Xu et al., 2000; Yang et al., 2000; Zhu et al., 2000). Wu et al. confirmed that Kir5.1 and Kir4.1 were co-expressed in the brainstem neural nuclei involved in the control of respiratory activity, such as the locus coeruleus (LC), parabrachial and Kolliker–Fuse (PBKF) nuclei, the ventrolateral medullary (VLM) area, and the nucleus of the solitary tract (NTS) (Wu et al., 2004). Therefore, the PCO_2_/pHi sensitive properties and localization of Kir4.1/5.1 channels suggest that they may be involved in the neuronal response to changes in PCO_2_ and pH in the cellular microenvironment.

The LC is a region in the pons that is chemically sensitive to CO_2_. Inhibition of Kir4.1/5.1 channels by hypercapnic acidosis can cause depolarization and lead to an increase in the excitability and firing frequency of more than 80% of the LC neurons (Wu et al., 2004; D'Adamo et al., 2011). Although the signaling between LC neurons and the medullary respiratory center remains controversial, increased excitability of LC neurons contributes to the regulation of respiratory activity during hypercapnia. Using mouse brain slices, researchers found that LC neurons in mice lacking *Kcnj16* had a reduced increase in firing frequency and a prolonged response time in response to intracellular acidification and hypercapnia compared with wildtype (WT) mice. This suggests that Kir5.1 is an important determinant of PCO_2_/pHi sensitivity in the LC neurons, is able to link CO_2_ levels in blood and cerebrospinal fluid to changes in neuronal activity, and may be involved in regulating respiratory responses during hypercapnic acidosis (Wu et al., 2004; D'Adamo et al., 2011).

However, another group of studies suggests otherwise. In 2011, Trapp et al. suggested that Kir5.1 knockout may not directly affect the sensitivity of central and peripheral respiratory chemoreceptors in mice. They used the Kir5.1 knockout mouse (Kir5.1^−/−^) model to observe changes in the ventilation response of Kir5.1^−/−^ mice at rest and in response to hypoxia and increased ambient CO_2_. Studies have found that Kir4.1 and Kir5.1 are expressed in both the respiratory center and peripheral carotid body (Wu et al., 2004; Yamamoto et al., 2008), but Kir5.1 knockout has no significant effect on central chemoreceptor sensitivity and carotid body function. The increases of respiratory frequency and respiratory depth in Kir5.1^−/−^ mice during hypercapnia and hypoxia were both lower than those in the WT group, which may be owing to abnormal signal transmission between peripheral chemoreceptors and the respiratory center; however, the mechanism is still unclear (Trapp et al., 2011). In addition, Puissant et al. observed a similar phenotype in SS^Kcnj16−/−^ rats, which showed a reduced ventilation response to hypercapnia and hypoxia (Puissant et al., 2019). These results demonstrate the important role of Kir5.1 channels in hypercapnic and hypoxic ventilation responses.

Other studies analyzed the exome data of 155 cases of sudden infant death syndrome (SIDS) and identified the *KCNJ16* R137S mutant. It was found that this mutant may cause disorders in signal reception in central respiratory chemoreceptors; thus, *KCNJ16* mutation may be a risk factor for SIDS (Neubauer et al., 2022). In conclusion, Kir5.1 knockout reduces the respiratory compensatory ability of the body to hypercapnia and hypoxia, which may be owing to abnormal signal transmission between peripheral respiratory chemoreceptors and central respiratory chemoreceptors or a signal reception disorder of the central chemoreceptors. The specific mechanism needs to be further studied in the future. In addition, the effects of metabolic acidosis caused by proximal tubular dysfunction in Kir5.1 knockout models on respiratory peripheral and central chemoreceptors should also be considered.

## 4 Research progress of Kir5.1 and human diseases

### 4.1 Kir5.1 and sensorineural deafness

Endolymph is the extracellular solution in the cochlear canal containing 150 mM K^+^ that exhibits a potential of approximately +80 mV relative to the adjacent perilymph, which represents the endocochlear potential (EP). The EP is a major driver of auditory signal transduction. External sounds are transmitted through the outer and middle ears, and then stimulate the cochlea of the inner ear. When the sound-driven vibration stimulates the basilar membrane, the hair bundles of hair cells are deflected, thus opening mechano-sensitive channels in the apical membrane of hair cells; then, the K^+^ in the endolymph flows into and stimulates hair cells. At the same time, the sound-driven vibration is converted into electrochemical signals, which are transmitted along the auditory nerve fibers to the auditory center, finally producing hearing. Subsequently, K^+^ is secreted by hair cells to the perilymph, further reabsorbed into support cells and spiral ligament fibrocytes, and finally transported to the stria vascularis; from the stria vascularis, K^+^ is re-secreted to the endolymph to maintain high K^+^ and EP in the endolymph, thus forming K^+^ circulation in the cochlea. Interference with any step of the K^+^ circulation pathway in the cochlea destroys the EP and leads to hearing impairment (Hibino et al., 2004b; Hibino and Kurachi, 2006; Lv et al., 2021).

In rats, Kir4.1 is abundantly expressed in the stria vascularis of the cochlea, whereas Kir5.1 is abundantly expressed in several specific types of fibrocytes (type II, IV, and V fibrocytes) of the spiral ligament in the cochlea lateral wall (Hibino et al., 2004b). Kir4.1 and Kir5.1 have completely different distribution positions in the cochlea, suggesting that they may function as homomeric channels in the cochlea. In addition, the expression of PSD-95 has also been detected in the cochlea. Kir5.1 subunits in fibrocytes are mostly expressed intracellularly, and Kir5.1 can combine with PSD-95 to form functional membrane channels (Tanemoto et al., 2002). Together with Kir4.1, Na^+^-K^+^-ATPase, Na^+^-K^+^-2Cl^−^-cotransporter, and other channels, Kir5.1 is involved in forming K^+^ circulation in the cochlea (Hibino et al., 2004b). Previous studies have demonstrated that mutations in the *KCNJ10* gene can cause sensorineural deafness (Bockenhauer et al., 2009; Reichold et al., 2010), but whether Kir5.1 dysfunction can cause hearing impairment is still controversial. Some studies have suggested that *Kcnj1*6 deletion in mice does not cause changes in hearing function, which may be related to compensation through other ion channels, such as KCNMA1, KCNQ4, and KCNE1 (Lv et al., 2021). However, in a group of disease types discovered by Schlingmann et al., eight patients with *KCNJ16* biallelic mutations at different positions all developed sensorineural deafness (Schlingmann et al., 2021). Therefore, the function of Kir5.1 in the cochlea and its role in hearing need to be further investigated.

### 4.2 Kir5.1 and cancer

Studies have found that potassium channels are not only involved in the control of the cell cycle and cell proliferation, but also in the processes of cell adhesion and migration, volume regulation, apoptosis, and angiogenesis, which are closely related to tumor biology, making potassium channels a potential therapeutic target for tumors (Pardo and Stuehmer, 2014; Comes et al., 2015). At present, studies on Kir5.1 and cancer mainly focus on the thyroid, pancreas, and prostate, among others, and the expression changes of Kir5.1 are not consistent in different glands during carcinogenesis (Table 1). In 2014, Ramos et al. found that Kir5.1, Kir4.1, and Kir4.2 were significantly expressed in the thyroid, but the function of Kir5.1 in the thyroid was unclear, although it was suggested to be involved in the transport of thyroglobulin (Ramos et al., 2015). Liu et al. analyzed mRNA datasets from 23 anaplastic thyroid cancer (ATC) samples and 24 normal samples and identified 55 differentially expressed genes (DEGs). Among them, the mRNA expression level of *KCNJ16* was low in ATC samples. Most of these DEGs are closely related to biological processes related to tumor progression. Therefore, identification of the key DEGs is of great significance for understanding the molecular mechanism of ATC (Liu et al., 2016). In addition, Kir5.1 is also expressed in both exocrine and endocrine regions of the pancreas (Liu et al., 2000; Pessia et al., 2001). In 2017, from GEO datasets of pancreatic ductal adenocarcinoma (PDAC) and normal pancreatic tissue samples, Jiang et al. found four differentially expressed potassium channels, and *KCNJ16* mRNA expression was found to be downregulated in PDAC samples (Jiang et al., 2017). In a 2022 study, RNA transcriptome profiles and clinical data analysis of patients with clear cell renal cell carcinoma (ccRCC) showed that *KCNJ16* expression was downregulated in patients with ccRCC, which was believed to be closely related to biological activities such as transmembrane transport and cell structure maintenance. Multiple ion channels are destroyed and altered during the development of ccRCC. Therefore, exploring the alteration of ion channels in the development of ccRCC may facilitate diagnosis and evaluation and inform the corresponding treatment (Zhu et al., 2022).

**TABLE 1:** Expression changes of Kir5.1 as a differentially expressed gene in human diseases.

Disease	Expression
Anaplastic thyroid cancer (ATC)	**↓** (Liu et al., 2016)
Pancreatic ductal adenocarcinoma (PDAC)	**↓** (Jiang et al., 2017)
Clear cell renal cell carcinoma (ccRCC)	**↓** (Zhu et al., 2022)
Parathyroid carcinoma (PCA)	**↑** (Adam et al., 2010)
Prostate cancer (PCa)	**↑** (Wu et al., 2020)
Hepatocellular carcinoma (HCC)	**↑** (Sun et al., 2022)
Preterm birth (PTB)	**↓** (Pereyra et al., 2019)
Non-familial Brugada syndrome (BrS)	*KCNJ16* Ser261Gly (Juang et al., 2014)
sudden infant death syndrome (SIDS)	*KCNJ16* R137S (Neubauer et al., 2022)

The up arrow indicates the upregulation of *KCNJ16* expression compared with the control group. The down arrow indicates the downregulation of *KCNJ16* expression compared with the control group.

Abnormally elevated *KCNJ16* expression may also lead to carcinogenesis. Through microarray transcriptome analysis of a parathyroid carcinoma (PCA) case and a biopsy from the same patient’s normal parathyroid gland, Adam et al. identified *KCNJ16* as one of the DEGs in PCA, and found that *KCNJ16* was upregulated in patients with PCA. These DEGs may serve as potential diagnostic markers for PCA (Adam et al., 2010). In addition, RT-qPCR on the mRNA of five prostate cancer cell lines and one normal prostate cell line showed that *KCNJ16* was upregulated in prostate cancer cell lines and was a DEG in patients with prostate cancer who had a Gleason score (GS) = 6 and GS ≥ 7. It is helpful to identify patients at high risk of biochemical recurrence after radical prostatectomy (Wu et al., 2020). Liu et al. applied the Significance Analysis of Microarray and Prediction Analysis of Microarray methods of GeneChip to screen out *KCNJ16* among 22 risk genes in the liver tissues of patients with chronic hepatitis and hepatocellular carcinoma (HCC) or cirrhosis. Further analysis revealed that *KCNJ16* mRNA was significantly upregulated in the HCC group. The detection of DEGs such as *KCNJ16* is helpful for the early diagnosis of cancer (Liu et al., 2014; Sun et al., 2022). These results suggest that *KCNJ16* can be used in genetic diagnostics for a variety of cancers. Early detection of lesions through genetic screening can provide earlier treatment opportunities and longer survival time for patients.

### 4.3 Kir5.1 and other diseases

In addition to the above human diseases, the abnormal expression of *KCNJ16* is also associated with diseases of other systems, such as the reproductive system, organ development, and the central nervous system. As early as 1999, it was demonstrated that Kir5.1 was expressed in the convoluted seminiferous tubules, spermatogonia, primary and secondary spermatocytes, and spermatozoa of rats (Salvatore et al., 1999). In 2021, Poli et al. used the *Kcnj16*-deleted mouse model to examine the possible role of Kir5.1 in the reproductive ability of mice. The results of the study found that Kir5.1 knockout mice had smaller testes, an increased proportion of sperm with abnormal morphology, and reduced fertility, compared with WT mice. Therefore, Kir5.1 channels play an important role in the development of mouse testes, the morphology of sperm flagella, and the maintenance of normal reproductive ability (Poli et al., 2021). The cattleyak (CY), a cross breed between cattle and yaks (YKs), has better adaptability to harsh environments, and its performance is much higher than that of YKs, including a larger size, higher quality meat, and higher milk production. However, CY females are fertile, whereas the males are sterile. In a 2020 study, researchers analyzed the epididymis transcriptomes of CY and YK, and found that *Kcnj16* was upregulated in CY. Thus, *Kcnj16* may be involved in the regulation of fluid and pH balances, and the regulation of luminal pH is important for spermatozoa maturation and storage in the caudal region of the epididymis (Liu et al., 2000; Park et al., 2017). Therefore, the upregulation of *Kcnj16* in CY may cause pH and luminal fluid imbalances, and eventually lead to male sterility in CY (Zhao et al., 2021). These studies provide new ideas for the research and diagnosis of human reproductive diseases.

In addition, Brugada syndrome (BrS) is an ion channelopathy associated with sudden cardiac death, and *SCN5A* is the most common mutated gene, accounting for 20%–25% of patients with BrS (Mizusawa and Wilde, 2012). Disease-targeted multigene sequencing was performed in 15 patients with non-familial BrS without *SCN5A* variants, and four mutated genes were identified, including *KCNJ16*. A variety of bioinformatic algorithms have been used for analysis to demonstrate that these mutants are harmful, and the discovery of these genes will help screen patients with non-familial BrS without *SCN5A* variants (Juang et al., 2014). In addition, through RNA sequencing analysis of the chorioamnion membranes of severe preterm and term fetuses, Pereyra et al. revealed 18 genes were downregulated in preterm infants, including *KCNJ16*. Abnormal ion channel function may be involved in the pathological process of preterm birth through pathways like Ca^2+^ signaling, smooth muscle contraction, and inflammatory response. These results help to establish the molecular landscape of preterm infants and improve the understanding of pathological mechanisms in preterm infants (Pereyra et al., 2019).

Moreover, it is known that loss of the transcriptional activation of the potassium channel Kir5.1 by hepatocyte nuclear factor 1 homeobox B drives autosomal-dominant tubulointerstitial kidney disease (Kompatscher et al., 2017). *Kcnj16* is frequently mutated in T-cell lymphoma in mice lacking the DNA mismatch repair gene *Mlh1*, but its function in T-cell lymphoma remains unclear (Daino et al., 2019). In addition, Kir5.1 is also expressed in oligodendrocytes and astrocytes in the central nervous system (Brasko et al., 2017; Patterson et al., 2021), and *Kcnj16* knockout produces audiogenic seizures in Dahl SS rats (Manis et al., 2021; Staruschenko et al., 2022). In conclusion, these multiple diseases caused in part or in whole by Kir5.1 dysfunction fully demonstrate its crucial role in the human body, and more studies are needed to explore its potential functions in the future.

## 5 Conclusion

This review mainly introduces the physiological characteristics of the inward-rectifying potassium channel Kir5.1, the pathophysiological processes related to Kir5.1, and its relationship with human diseases (Table 2). Kir5.1 not only has pHi sensitivity, but also acts as a potassium sensor, allowing its involvement in both the regulation of electrolyte homeostasis and acid-base balance in the kidneys and in the formation of the salt-sensitive phenotype in Dahl SS rats. Kir5.1 is expressed in both central and peripheral respiratory chemoreceptors and can give central respiratory chemoreceptor neurons the ability to sense PCO_2_ and pHi and regulate respiration by changing their firing frequency. Kir5.1 dysfunction reduces the ability of the body to compensate for hypoxia and hypercapnia. *KCNJ16* biallelic mutations can lead to sensorineural deafness, but the role of Kir5.1 in auditory system requires further research. Meanwhile, several genomic analyses have shown that *KCNJ16* is a DEG in a variety of cancers, such as ATC, PDAC, and HCC. In addition, potential roles of Kir5.1 in the reproductive system and in organ development have been identified.

**TABLE 2:** The localization and involvement of Kir5.1 in physiological processes and Kir5.1 dysfunction.

Localization	Affected physiological processes	Kir5.1 dysfunction
Epithelial cells in proximal tubules, TAL, DCT, and principal cells in CCD	Participate in the regulation of systemic electrolyte homeostasis; involved in the formation of the salt-sensitive phenotype	Metabolic acidosis and hypokalemia; loss of salt-sensitive phenotype in Dahl SS rats
Carotid body; brainstem neural nuclei such as LC, PBKF, VLM, and NTS	Give central respiratory chemoreceptor neurons the ability to sense PCO_2_ and pHi and regulate respiration by changing their firing frequency	Decreased respiratory compensatory capacity against hypercapnia and hypoxia
Type II, IV, and V fibrocytes of the spiral ligament in the cochlea lateral wall	Involved in the K^+^ circulation in the cochlea and form the EP	Sensorineural deafness
Seminiferous tubules, spermatogonia, primary and secondary spermatocytes, and spermatozoa	Involved in spermatogenesis, development, and maturation	Smaller testes, an increased proportion of abnormal morphology sperm, and reduced fertility
Thyroid follicular cells	May be involved in the transport of thyroglobulin	/
Exocrine and endocrine regions of the pancreas	/	/

Abbreviations: TAL, thick ascending limb; DCT, distal convoluted tubule; CCD, cortical collecting duct; SS, salt-sensitive; LC, locus coeruleus; PBKF, parabrachial and Kolliker-Fuse; VLM, ventrolateral medullary; NTS, nucleus of solitary tract; pHi, intracellular pH; EP, endocochlear potential.

There are several shortcomings in the existing research. In contrast to the large number of studies focused on the kidneys and brain, previous studies on Kir5.1 rarely focus on its functions in the thyroid or pancreas. In 2022, during high-throughput screening of multiple compounds, McClenahan et al. discovered and validated VU6036720, the most potent Kir4.1/5.1 channel-selective inhibitor reported to date. The lack of studies on specific inhibitors of Kir4.1/5.1 channels has limited further investigation into the physiological function of Kir5.1 (McClenahan et al., 2022). In addition, future studies that focus on the regulatory mechanisms of this ion channel will facilitate the potential use of Kir5.1 as a valuable targeted therapy or regulatory strategy, and increase our understanding of this unique Kir channel family member.
